# Exploring YouTube Videos About Anorexia Nervosa on the Basis of Reliability, Popularity, and Contributions of Healthcare Professionals: A Cross-Sectional Study

**DOI:** 10.7759/cureus.48095

**Published:** 2023-11-01

**Authors:** Ananthakrishnan Suresh, Lalitha Lalithya Pallempati, Palak Saxena, Ayesha Ansari, Radhika Bassi, Ajita Bhandari

**Affiliations:** 1 Internal Medicine, Calicut Government Medical College, Kozhikode, Kerala, IND; 2 Internal Medicine, Kamineni Academy of Medical Sciences and Research Centre, LB Nagar, Hyderabad, IND; 3 Internal Medicine, Dr. Sampurnanand Medical College, Jodhpur, IND; 4 Anaesthesia, D.Y. Patil Medical College, Kolhapur, IND; 5 Pediatrics, Ross University School of Medicine, Bridgetown, BRB; 6 Pediatrics, Lumbini Medical College and Teaching Hospital, Palpa, NPL

**Keywords:** healthcare professionals, quality assessment, symptoms, treatment, content analysis, perception of reliability, youtube, eating disorder, anorexia nervosa

## Abstract

Background: Anorexia nervosa, an eating disorder, is characterized by a distorted body image, intense fear of gaining weight, and self-imposed starvation. The aim of this study is to analyze type of information as well as its quality and reliability on YouTube about anorexia nervosa.

Methodology: A cross-sectional observational study was conducted on 59 Youtube videos using anorexia nervosa-related keywords in June 2023. The characteristics of the YouTube videos, such as the language of the information, the time of upload, and the qualifications of the uploaders, were recorded. The content and quality of 59 videos were assessed using the Global Quality Score (GQS) and reliability grading systems.

Results: The videos accumulated a total of 256,602 likes, 5,644 dislikes, and 17,761 comments. Treatment-related content accounted for 81.36% of the videos, while descriptions of symptoms comprised 79.66%. Doctors contributed to 18.6% of the total uploads, making them the second-largest group of uploaders after the 'Other' category. The median reliability score for doctors is 4, the same as the hospital healthcare organization. This indicates that the perceived reliability of doctors as a source of information is as high as that of hospital healthcare organizations.

Conclusions: In conclusion, this study highlights the importance of critically evaluating information on anorexia nervosa videos on YouTube. Despite variations in popularity, the overall quality and reliability remained consistent. Doctors were perceived as reliable sources of information, comparable to hospital healthcare organizations. Ensuring accurate and trustworthy content is crucial for supporting those affected by anorexia nervosa and promoting reliable information to the public.

## Introduction

Anorexia nervosa is a psychosomatic eating disorder associated with body image preoccupation and a morbid fear of gaining weight [1]. The disorder usually affects more females than males. Broadly, anorexia nervosa has been classified into two types: restrictive type and binge-eating type. Patients with the restrictive type are characterized by peculiar food-handling behavior that involves restricting their food intake and indulging in vigorous physical activities to maintain their body shape [1]. Binge eating, however, is characterized by episodes of binge eating followed by post-binge anguish resulting in compensatory purging or vomiting [1]. Eating disorders are complex conditions that frequently occur with other ailments and have multifaceted mechanisms like affected cognitive control, reward processing, and affective processing [2]. According to new Diagnostic and Statistical Manual of Mental Disorders (DSM-5) criteria, patients with anorexia nervosa are underweight for their age. Complications include menstrual abnormalities; cardiovascular consequences, most commonly sinus bradycardia; hyponatremia; hypokalemia; hypoglycemia; leukopenia; CNS atrophy; and gastrointestinal tract (GIT) disorders. This can lead to a high mortality rate in patients with anorexia nervosa [3].

The increasing influence of social media and multimedia acts as a powerful instrument in shaping the behavior and attitudes of people. YouTube is an American video-sharing social media platform that has been used for distributing and watching content, both relevant and irrelevant, by all kinds of people. Social media influencers, actors, religious leaders, doctors, hospitals, educational institutes, etc., all use YouTube for sharing information, promotions, and entertainment. An individual’s body image and self-esteem are generally negatively impacted by the media. The media presents a variety of strict messages favoring thin bodies, which are frequently unattainable for most women. As a result, they experience body dissatisfaction [4].

Usually, many individuals suffering from any disorder first resort to Google searches, YouTube videos, or other social media information before consulting qualified doctors. Online searches help in increasing literacy and awareness and promoting health and recovery. These advantages, however, hinge on the authenticity, readability, design, content, and structure of videos. Uneducated or illiterate people are unable to comprehend the information and often become misguided [5]. Thus, our study here is an attempt to assess the quality, and reliability of videos available on YouTube.

## Materials and methods

This is a cross-sectional observational study that utilized publicly available videos from the popular online video-sharing platform YouTube. Six keywords related to the topic of anorexia nervosa were used in a YouTube search. These were: "Anorexia nervosa", "Anorexia nervosa treatment" , "Anorexia nervosa care plan" , "Anorexia nervosa recovery", "Anorexia nervosa therapy" and "Anorexia". Since the study did not involve human participants, no ethical approval was needed from the ethics committee.

Inclusion criteria

The requirements of the inclusion criteria were as follows: The videos should be relevant to the topic; the videos’ content should contain a discussion on symptoms, etiology, preventive measures, treatment options, recovery, rehabilitation, support groups, mortality, patient experiences; whether a video includes any promotional material must be mentioned; the video source (i.e., a doctor, a health organization, news channels, or others) should be noted; the video must be more than one minute long but no longer than 20 minutes; and it must be in English exclusively. Any video that failed to satisfy the inclusion criteria was excluded.

A total of 70 videos were reviewed, and their descriptive features such as the number of likes, dislikes, views,comments, duration, upload date, and demographic features such as source and uploader (e.g., a doctor, a health organization, news channels, or others) were noted. The content of the videos was evaluated for quality with the GQS score and reliability score (DISCERN) [6,7]. When the GQS tool was used, consideration was given to the significance of the information provided, the quantity of pertinent material, the thoroughness of the description and explanations, and the flow of the video. A score of 5, the highest possible, denotes great quality and fluidity. The information’s accuracy and dependability were evaluated with the DISCERN score.

To help researchers enter their findings, the responses were collected via Google Forms and subsequently extracted into an Excel spreadsheet. SPSS software was used to carry out the statistical analysis.

## Results

A total of 70 videos were assess, and finally, after inclusion-exclusion criteria and removal of duplicates, 59 YouTube videos were taken into consideration. The total number of views, likes, dislikes, and comments were 12,641,436, 256,602, 5,644, and 17,761, respectively.

Table 1 shows the characteristics of the YouTube videos analyzed. A large number of videos (42 (71.2%)) were uploaded more than a year ago. Only 11 (18.6%) YouTube videos were uploaded by doctors and seven (11.9%) by hospital and healthcare organizations. 25 (42.4%) of them were uploaded by unverified uploaders.

**Table 1:** Characteristics of YouTube videos analysed

Parameter	n (%)
Time since uploaded	
More than a week to six months (7-180 days old)	10 (16.9%)
More than six months to last one year (180-365 days)	07 (11.9%)
More than one year (> 365 days)	42 (71.2%)
Popularity	
Total no. of views	12,641,436
Total no. of likes	256,602
Total no. of dislikes	5,644
Total no. of comments	17,761
Type of uploader	
Doctor	11 (18.6%)
Hospital/healthcare organization	07 (11.9%)
News channel	09 (15.3%)
Patient	07 (11.9%)
Other	25 (42.4%)

Table 2 shows the type of information about anorexia in the YouTube videos. About 48 (81.36 %) of the videos discussed the treatment and 47 (79.66 %) talked about the symptoms. Also, 35 (59.32 %) videos had information about support groups and 20 (33.9%) videos had information about investigations. Very few videos had promotional content (3 (3.39%)) or parents of the patient sharing their experiences with their family members (6 (10.17%)). 

**Table 2:** Type of information about anorexia in the YouTube videos

Type of information	n (%)
Information about cause/etiology	30 (50.85%)
Information about investigations/test	20 (33.9%)
Information about prevention	8 (13.56%)
Information about treatment	48 (81.36%)
Information about mortality	21 (35.59%)
Information about rehabilitation	35 (59.32%)
Information about support groups	18 (30.51%)
Information about patients sharing their own experience	25 (42.37%)
Information about parents sharing experience with their family members	6 (10.17%)
The post has a promotional content by pharmaceutical company or by doctors	2 (3.39%)

Table 3 shows the comparison of GQS, reliability score, and Video Power Index (VPI) based on the uploader. The VPI of YouTube videos uploaded by news agencies (314.06 (20.05, 820.82)) and patients suffering from anorexia (252.31 (11.58, 2038.13)) is statistically significantly higher than videos uploaded by doctors (3.69 (1.03, 38.21)) and hospitals (13.21 (6.97, 19.83)), signifying higher reach and user interaction of YouTube videos uploaded by news channels and patients of anorexia. However, there is no significant difference (p>0.05) in the quality and reliability of information about anorexia based on the type of uploader. 

**Table 3:** Comparison of GQS, reliability score and VPI based on type of uploader GQS: Global Quality Score, VPI: Video Power Index; IQ: Interquartile range p <0.05 is significant

	Doctors (n=11)	Hospital/healthcare organization (n=07)	News channel (n=09)	Patient (n=07)	Other (n=25)	P value & test used
	Median (IQ1, IQ3)	Median (IQ1, IQ3)	Median (IQ1, IQ3)	Median (IQ1, IQ3)	Median (IQ1, IQ3)	Test Used: Kruskal-Wallis Test
VPI	3.69 (1.03, 38.21)	13.21 (6.97, 19.83)	314.06 (20.05, 820.82)	252.31 (11.58, 2038.13)	27.16 (2.83, 139.5)	P-value = 0.015
GQS	4 (3, 5)	4 (4, 5)	4 (2, 4)	4 (3, 5)	4 (3, 4)	P-value = 0.351
Reliability Score	4 (3, 5)	4 (3, 5)	3 (2, 4.5)	4 (2, 5)	3 (2, 4)	P-value = 0.312

## Discussion

Anorexia nervosa is a psychiatric condition predominantly seen at the beginning of adolescence and can have a progressively detrimental impact on patients’ health. It is associated with weight loss due to malnourishment [8]. The emergence of health-related channels on social media, particularly YouTube, has significantly increased awareness of anorexia nervosa. The availability of such information along with diagnosis, prevention, and treatment plans is readily available in a concise manner. However, the very nature of social media is concerning due to the presence of misinformation; promotions of pharmaceuticals; and most importantly, the contributions of individuals who do not belong to the healthcare community [9]. In this study, we have observed that the information shared in more than 70% of the evaluated YouTube videos was not made by healthcare providers.

The easy access to YouTube has raised the cognizance surrounding anorexia nervosa. The discussion of symptoms, treatment, and knowledge of support groups has increased as a result. In this study, it was found that the majority of the videos 47 (79.66%) discussed the symptoms of anorexia nervosa and 48 (81.36%) of the videos focused on treatment. The presence of vloggers and people who have experienced anorexia nervosa firsthand has helped others with the recognition of this disorder [10]. Hence, social media platforms play an important role in creating awareness of diseases, especially related to mental health.

However, the authenticity of an individual or organization spreading health-related information on social media must be questioned due to the double-edged nature of online platforms. The presence of uploaders without proper qualifications can lead to the spread of medical misinformation and cause detrimental effects on the population as a whole [11].

In this study, it was discovered that 25 (42.4%) of the videos were uploaded by other sources. It was also found that two (3.39%) videos were uploaded as promotional content. Such videos raise concerns due to the surge of pro-anorexia content, especially among the youth [12]. Youth engagement in social media content due to relevancy rather than accuracy of information requires close monitoring.

However, the YouTube metrics, including views, VPI, reliability score, and GQS, however, play a crucial role in determining the reach and steadfastness of the content. While uploading medical information, it is highly recommended to promote videos with higher quality control and information checks [12]. Proper regulations of content uploaded on social media should be implemented. This should be extended to all health-related videos in order to provide a safe space and conscientious data for the improvement of health in our population, especially young adults.

Limitations

The total number of videos evaluated was merely 70. There may be superior-quality videos with a duration of more than 20 minutes; however, in this study, only videos with a duration of one to 20 minutes were included. In this study, only the quality of videos from a subjective point of view was assessed, and the geographic variations of the information discussed in a video were not taken into consideration. Hence, for the same video, there may be interobserver differences.

## Conclusions

From this study, we concluded that the information uploaded on social media needs to be verified by properly qualified people such as doctors and/or healthcare organizations. This information should be easy to understand, with a high discernment and GQS score enabling the general population to comprehend the medical condition. These videos should also contain information on how one can receive a diagnosis and treatment from a doctor rather than utilizing the knowledge to self-diagnose.
